# Exosomal Long Non-Coding RNA ANCR Mediates Drug Resistance in Osteosarcoma

**DOI:** 10.3389/fonc.2021.735254

**Published:** 2022-01-12

**Authors:** Xin Hu, Yang Wen, Lin-yun Tan, Jie Wang, Fan Tang, Yi-tian Wang, Chuan-xi Zheng, Yu-qi Zhang, Tao-jun Gong, Li Min

**Affiliations:** Orthopedic Research Institute, Department of Orthopedics, West China Hospital, Sichuan University, Chengdu, China

**Keywords:** osteosarcoma, drug resistance, lncRNA, ANCR, exosomes

## Abstract

Osteosarcoma (OS) is rare cancer with bimodal age distribution with peaks observed in children and young adults. Typically, OS is treated with pre-surgery neoadjuvant therapy, surgical excision, and post-surgery chemotherapy. However, the efficacy of treatment on disease prognosis and objective response is not currently optimal, often resulting in drug resistance; in turn, highlighting the need to understand mechanisms driving resistance to therapy in OS patients. Using Doxycycline (Dox)-sensitive and resistant variants of OS cells lines KHOS and U2OS, we found that the resistant variants KHOS-DR and U2OS-DR have significantly higher *in vitro* proliferation. Treating the Dox-sensitive KHOS/U2OS cells with exosomes isolated from KHOS-DR/U2OS-DR made them resistant to treatment with Dox *in vitro* and *in vivo* and enhanced tumor growth and progression, while decreasing overall survival. Expression of the long non-coding RNA (lncRNA) ANCR was significantly higher in the KHOS-DR and U2OS-DR variants. SiRNA-mediated knockdown of ANCR decreased *in vitro* proliferation, while increasing sensitivity to Dox treatment in the KHOS-DR/U2OS-DR cells. Expression of the exosomal lncRNA ANCR was critical for drug resistance and OS tumor progression in xenografts and was correlated to resistance to Adriamycin and overall survival is patients with OS. These results establish lncRNA ANCR as a critical mediator of resistance to therapy in OS patients, highlighting it as a potential therapeutic target in OS patients.

## Introduction

Osteosarcoma (OS) is rare cancer but is the most common bone neoplasm (1). It originates as a localized aggressive tumor with a high incidence of metastasis to distant organs (1, 2). The bone microenvironment in which OS grows is a dynamic and specialized compartment composed of the malignant bone cells (osteocytes, osteoclasts, and osteoblasts), vascular cells, stromal cells (mesenchymal stem cells and fibroblasts), mineralized extracellular matrix, and immune cells (3–5). Recent studies have highlighted the importance of the tumor microenvironment (TME) in OS pathogenesis and progression (6–8). OS cells can control the balance between M1 and M2 macrophages, in turn regulating T cell response within the TME *via* programmed cell death protein 1 (PD-1) and programmed death ligand-1 (PD-L1) (8). Indeed, different studies using mice models of OS or clinical trials in OS patients have shown objective response on survival and metastatic progression using immune checkpoint inhibitors (ICRs) (9–15). Expression of both PD-L1 and PD-1 have been shown to negatively correlate with prognosis in OS patients (12).

Pre-surgical neoadjuvant therapy, followed by surgical excision, and post-surgery chemotherapy with high-dose methotrexate, ifosfamide, and etoposide are typically used to treat patients with OS (16–19). However, such treatments are not efficacious (1, 20–22), often resulting in resistance to treatment. This highlights the requirement for the identification of mechanisms underlying resistance to therapy in OS patients.

Doxorubicin (Dox) is one of the leading and first-line drugs used as chemotherapy in osteosarcoma (OS). However, 40% to 45% of high-grade OS patients are resistant, or only partially responsive, to doxorubicin (23–25). Chemoresistance to DOX is mediated by different mechanisms, including increased drug inactivation, enhanced efflux or attenuated drug influx, improved activity of the DNA repair machinery, cancer stem cells, and enrichment of pre-survival pathways (24–26). However, it has been shown that the overexpression of the drug efflux transporter ATP binding cassette transporter B1 (ABCB1) or P-glycoprotein (Pgp) is central to resistance to Dox (19, 27–30). ABCB1 induces DOX efflux preventing its intracellular accumulation and cytotoxicity. Indeed, ABCB1 is a robust prognostic biomarker to predict sensitivity and outcome to first-line chemotherapy in OS patients. The mechanistic details for resistance to Dox though remain elusive.

Mounting evidence indicate that cancer exosomes function as mediators of drug resistance (31). In addition, both lncRNAs and microRNAs have been shown to be key determinants of OS pathogenesis, disease progression, and chemoresistance (32–36). However, whether plasma-derived or tumor-derived exosomes regulate Dox resistance in OS patients has not been investigated. Hence, in the current study we initially determined if exosomes derived from Dox-resistant OS cell lines can drive resistance to Dox treatment in native Dox-sensitive cell lines. Given that the expression of lncRNA ANCR has been shown to be overexpressed in OS and correlates to disease progression (37), we next determined in lncRNA ANCR expression was different in exosomes derived from Dox-sensitive and Dox-resistant OS cell lines. Our results show that exosomes derived from Dox-resistant OS cell lines can drive Dox-resistance in Dox-sensitive cell lines *in vitro* and enhance tumor progression *in vivo*. In addition, expression of the lncRNA ANCR was higher in exosomes derived from Dox-resistant OS cell lines and its downregulation decreased cell proliferation *in vitro* and decreased disease progression *in vivo*.

## Materials And Methods

### Patients and Sample Processing

Osteosarcoma patients without metastasis or recurrence in 5 years post-surgery are defined as chemosensitive. Osteosarcoma patients with metastasis or recurrence within 5 years post-surgery are defined as chemoresistant. Peripheral blood samples from osteosarcoma patients chemoresistant and chemosensitive to Adriamycin (n=10 each) were obtained retrospectively after pathological diagnosis of disease, and after adjuvant/neoadjuvant chemotherapy and surgery. Blood serum were extracted and frozen in a -80° refrigerator for future usage. Blood were collected under a protocol approved by the Institutional Ethics Committee of West China Hospital (Chengdu, China, Approval No. 2019-122), and the study protocol adhered to the guidelines stipulated in the World Medical Association Declaration of Helsinki. All patients involved in the study provided signed informed consent.

### Cell Culture and Transfection

The Dox-sensitive OS cell lines KHOS and U2OS and the Dox-resistant OS cell lines, KHOS-DR and U2OS-DR were obtained from the Sarcoma Biology Laboratory, Department of Orthopaedic Surgery, Massachusetts General Hospital and Harvard Medical School, and grown in Dulbecco’s Modified Eagle Medium (DMEM, Invitrogen, Carlsbad, CA, USA). The medium was supplemented with 10% fetal bovine serum (FBS, Invitrogen, Carlsbad, CA, USA) and 1% penicillin-streptomycin solution. Cells were incubated in a 5% CO_2_ environment at 37°C. Where indicated, exponentially growing cells were transfected with either a non-silencing siRNA control or siRNA targeting lncRNA ANCR (final concentration 20 nM) (Ribobio, Guangzhou, China) using Lipofectamine 3000 (Thermo Fisher Scientific, Carlsbad, CA, USA).

### Cell Growth Assay

Cells were grown in clear-bottom 96-well plates and treated as indicated in figure legends. Cell proliferation was analyzed using a CCK-8 assay kit (Sigma-Aldrich, St. Louis, MO) as per the manufacturer’s recommendations. The absorbance was measured at 450 nm and post-measurement corrected by subtracting absorbance at the reference wavelength. The data plotted in the figures are expressed as relative optical density (OD).

### MTT Assay for Cell Survival

Cells were grown in clear-bottom 96-well plates and treated as indicated in figure legends. Cell proliferation was analyzed using a mitochondrial colorimetric assay (MTT assay, Sigma-Aldrich, St. Louis, MO) as per the manufacturer’s recommendations. The absorbance was measured at 570 nm and post-measurement corrected by subtracting absorbance at the reference wavelength of 690 nm. The data plotted in the figures are expressed as relative optical density (OD).

### Western Blot Analysis

Whole cells or exosomes were lysed using RIPA buffer. Protein samples were resolved by SDS-PAGE. Primary antibodies used were anti-CD9 (Catalogue # 223052), anti-CD63 (Catalogue # ab68418), anti-CD81 (Catalogue # ab109201), anti-GM130 (Catalogue # ab52649), anti-Calreticulin (Catalogue # ab92516), and anti-Alix (Catalogue # 117600) (all antibodies were purchased from Abcam, Waltham MA, USA and used at 1:1000 dilution). GAPDH was used as the loading control.

### Immunofluorescence Staining

Exosomes were labeled with PKH67 using a green fluorescent linker kit (Abcam). Immunofluorescence was done using previously described protocol (38). Antibodies used were anti-Transferrin (Catalogue # ab82411; 1:250 dilution). Post-incubation with primary antibody, slides were washed with PBS and incubated with secondary antibodies and DAPI in room temperature. Images were obtained using an Olympus FV1000 confocal (Olympus) microscope.

### Isolation of Exosomes

Exosomes were isolated from the indicated OS cell lines and peripheral blood from OS patients using the Total Exosome Isolation Reagent kit (ThermoFisher Scientific, Carlsbad, CA, USA) strictly following the manufacturer’s protocol. Isolated exosomes were immediately processed for RNA and protein isolation.

### Transmission Electron Microscope and Determination of Size Distribution of Exosomes

For transmission electron microscope, the exosome samples were fixed using 2.5% glutaraldehyde at 4°C overnight. Afterward, 10 μl of exosomes solution was placed on the copper grids for 10 min, following by adding 10 μl of 2% phosphotungstic acid solution and staining for 2 min at room temperature. After remove excess staining solution and allow the copper grids dry at room temperature, the exosome samples was observed under transmission electron microscope at 120 kV.

For determination of size distribution of exosomes, the purified exosomes was diluted in sterile PBS solution and filtered using 0.22 μm filter. Subsequently, the size distribution and concentration of exosomes samples were measured using nanoparticle tracking analysis (NTA) with NanoSight NS300 (Malvern Instruments Ltd.; Malvern, UK).

### Isolation of Exosomal RNA and Protein

Protein and RNA from enriched exosomes were isolated using the Total Exosome RNA & Protein Isolation kit (Thermo Fisher Scientific, Carlsbad, CA, USA) using the manufacturer’s protocol.

### Real Time Quantitiative Polymerase Chain Reaction (RT-qPCR)

SuperScript III reverse Transcriptase [Thermo Fisher Scientific, Carlsbad, CA, USA)] was used for cDNA synthesis from the exosomal RNA. RT-qPCR reactions were set up using KAPA SYBR FAST (KAPA BIOSYSTEMS, Wilmington, USA). The primers used were: *DANCR* – forward primer: 5′- GACATTTCCTGAGTCGTCTTCGAACGGAC -3′; reverse primer: 5′- TAGTGCGATTTAGAGCTGTACAAGTTTC -3′; Raw Ct values were normalized to 18s rRNA (forward primer: 5′- ACACGGACAGGATTGACAGA-3′; reverse primer: 5′-GGACATCTAAGGGCATCACA -3′) and changes in expression were calculated using the 2^-ΔΔCt^ method.

### Spontaneous Xenograft Metastasis Model

All procedures were performed under the approval of the Laboratory Animal Welfare and Ethics Committee of the West China Hospital of Sichuan University. KHOS/U2OS cells (5×10^5^), parental or pre-treated with exosomes isolated from KHOS-DR/U2OS-DR cells were trypsinized, washed with PBS, and resuspended in a 1:1 solution of PBS and Matrigel (phenol red-free and reduced growth factors, BD Biosciences) and injected into the lateral tail vein of 6-weeks old female nude mice (n = 20 each). Ten mice were kept as such without any injection of U2OS cells (sham control). The mice were randomly divided into two experimental groups each (n = 10/group) – the control groups (KHOS/U2OS – Dox, and KHOS/U2OS + Exosomes – Dox) received no treatment, whereas the treatment groups (KHOS/U2OS + Dox, and KHOS/U2OS + Exosomes + Dox) received 1 mg/kg of Dox orally once every 3 days for 4 weeks. Bodyweight and tumor size was measured every 7 days. The mice (n=5/group) were sacrificed 4 weeks post-inoculation. The other 5 mice/groups were kept until all mice in one group died, and the data was used for survival rate analysis. The tumors were surgically removed and processed similarly to patient samples, and fixed in 4% polyformaldehyde, and stored at -80°C.

### Hematoxylin & Eosin (H&E) and Immunohistochemistry (IHC) Staining

The tissues were fixed and cut into 4 µm sections. The slides were either processed for H&E staining using routine methodologies or treated for antigen retrieval and incubated with primary antibodies (Osteocalcin, 1:200; Catalogue # ab93876, Abcam, Waltham, MA, USA) overnight at 4°C. Images were obtained using an Olympus light microscope.

### Statistical Analysis

Quantitative data were expressed as the mean ± standard deviation (SD). The student’s t-test was used for two-group comparison, and the differences between more than two groups were performed using ANOVA. Kaplan-Meier curves were computed to analyze survival rates and the Log-rank (Mantel-Cox) test to evaluate statistical significance. P<0.05 was considered statistically significant.

## Results

### Drug-Resistant OS Cell Lines Exhibit Higher *In Vitro* Proliferation

**Figure 1:**
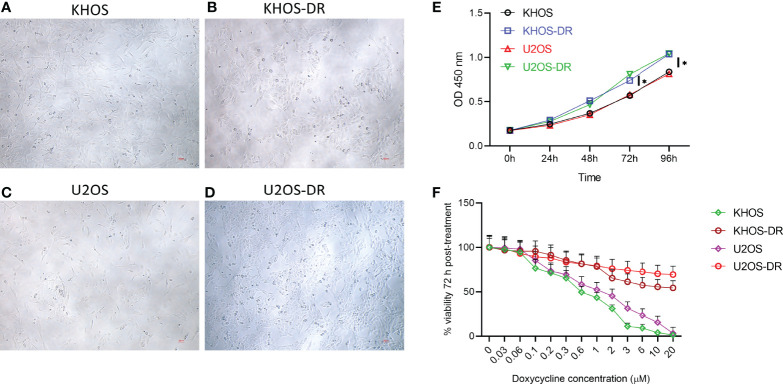
Drug-resistant osteosarcoma (OS) cell lines exhibit higher growth rate *in vitro*. **(A–D)** Photomicrographs of Dox-sensitive OS cells line KHOS and U2OS and their corresponding resistant variants, KHOS-DR and U2OS-DR, respectively. Scale bar, 200 µm. There was no robust difference in the morphology of the different cell types. **(E)** Relative growth of the Dox-sensitive KHOS and U2OS cells lines and the Dox-resistant KHOS-DR and U2OS-DR were quantified using CCK8 assay. U2OS-DR and KHOS-DR cell lines had significantly higher growth over 4 days compared to U2OS and KHOS, respectively. *P < 0.05. **(F)** Indicated cells were treated with different concentration of Dox for 72 h and cell viability was measured. For both **(E, F)**, data is representative of three independent experiments, each measured in triplicate. Error bars, SD.

We initially determined if there is any difference in gross morphology between the Dox-sensitive OS cells line KHOS and U2OS and their corresponding resistant variants, KHOS-DR and U2OS-DR. Photomicrographs did not reveal any robust difference in their appearance, even though the drug resistant variants appeared to be more compactly packed together (**Figures 1A–D**). The drug resistant KHOS-DR and U2OS-DR cells exhibited significantly higher growth rate at 72 h and 96h, when compared to KHOS and U2OS cells, respectively (**Figure 1E**). We next confirmed the Dox-sensitivity and resistance in these cell variants. All variants were treated with increasing concentration of Dox for 72h. The KHOS-DR and U2OS-DR were resistant to Dox as expected (**Figure 1F**).

### Exosomes Derived From Dox-Resistant Cells Can Drive *In Vitro* Chemoresistance to Dox in Dox-Sensitive Cells

**Figure 2:**
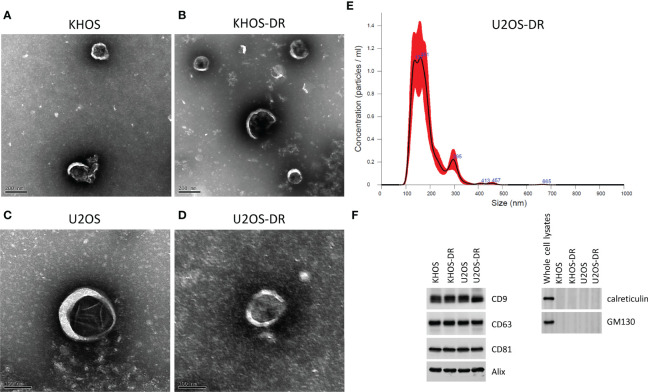
Validation of exosome purification. **(A–D)** Scanning electron micrograph of exosomes purified from KHOS, KHOS-DR, U2OS, and U2OS-DR. Scale bar, 200 nm. N = 3 biological samples. **(E)** Representative nanoparticle tracking analysis of exosomes purified from U2OS-DR. N = 3 biological samples. Similar results were obtained from the KHOS, KHOS-DR, and U20S. **(F)** Western blot analysis of control exosome markers CD9, CD63, CD81, Calreticulin and GM130. Alix was used as a loading control. Western blots have been cropped for clarity and conciseness. N = 3 technical replicates.

We next wanted to determine if exosomal content or architecture are different in Dox-sensitive and resistant OS cell lines. Exosomes were isolated from KHOS, KHOS-DR, U2OS, and U2OS-DR cells. Transmission electron microscopy did not reveal any difference between exosomal architecture between the Dox-sensitive (**Figure 2A**) and Dox-resistant (**Figures 2B, D**) cells. Nanoparticle tracking analysis of exosomes purified from these cells revealed a median size range of 150-200 nm (**Figure 2C**, and *data not shown*). Analysis of exosome markers CD9, CD63, and CD81 revealed equivalent expression in exosomes isolated from all cell variants (**Figure 2D**). The lack of western blots of ER and Golgi markers GM130 and Calreticulin showed qualified exosome extracts have been prepared and used in our experiments with additional controls of whole cell extracts of KHOS cell line showing our antibodies are functional (**Figure 2F**).

**Figure 3:**
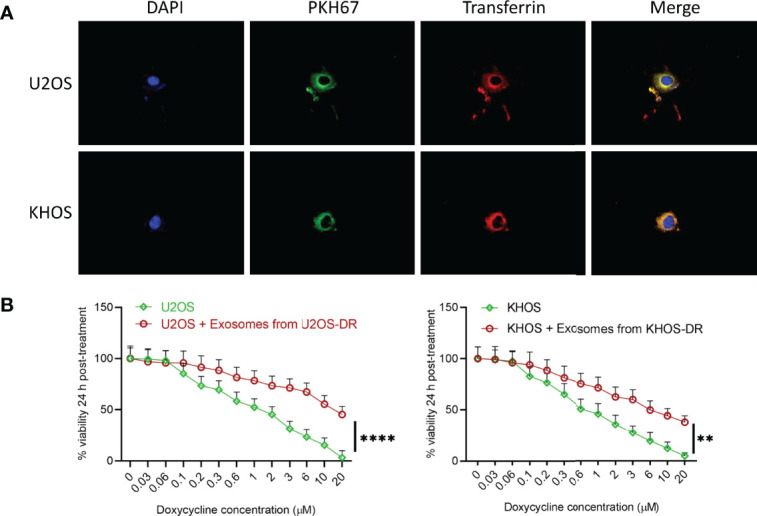
Exosomes from KHOS-DR/U2OS-DR induce *in vitro* drug resistance in U2OS cells. **(A)** PKH67-labeled exosomes from KHOS-DR/U2OS-DR cells (green) were rapidly internalized into early endosomes labeled with Texas Red transferrin (yellow indicates colocalization of green and red). DAPI, nucleus. **(B)** Post-internalization of exosomes isolated from KHOS-DR/U20S-DR cells, the KHOS/U2OS cells were updated treated with indicated concentration of Dox for 24 h and cell viability was measured. Exosomes from KHOS-DR/U2OS-DR induced resistance to Dox in the KHOS/U2OS cells (IC_50_ 1.32 ± 0.12 µM to 15.67 ± .76 µM). Data is representative of three independent experiments, each measured in triplicate. Error bars, SD; **P < 0.01, ****P < 0.0001.

We next determined if exosomes isolated from the Dox-resistant KHOS/U2OS cells could induce drug-resistance in Dox-sensitive cells. Dox-sensitive KHOS/U2OS cells were treated with exosomes isolated from Dox-resistant KHOS-DR/U2OS-DR cells. The exosomes isolated from KHOS-DR/U2OS-DR cells were labeled with PKH67 to ensure they were being internalized in the KHOS/U2OS cells. PKH67-labeled exosomes from KHOS-DR/U2OS-DR cells were rapidly internalized into early endosomes labeled with transferrin (**Figure 3A**). Post-internalization of exosomes treatment of the Dox-sensitive KHOS/U2OS cells induced chemoresistance in the normally sensitive KHOS/U2OS cells (IC_50_ 1.32 ± 0.12 µM to 15.67 ± .76 µM) (**Figure 3B**). Taken together these results indicated that exosomes from chemoresistant KHOS-DR/U2OS-DR confers doxorubicin resistance to KHOS/U2OS cells.

### Exosomes From U2OS-DR Induced Drug Resistance and Enhances OS Tumor Progression *In Vivo*

**Figure 4:**
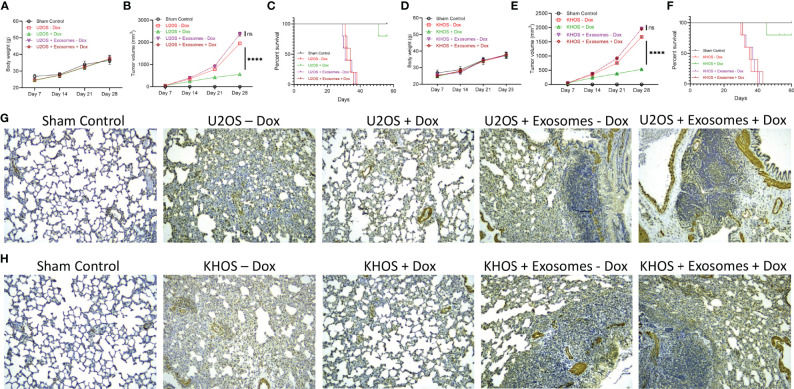
Exosomes from KHOS-DR/U2OS-DR induce drug resistance and enhances OS tumor progression *in vivo*. Nude mice were injected with Matrigel (sham control) (n=10), KHOS/U2OS cells (5 x 10^5^ in Matrigel; n = 20), and KHOS/U2OS cells pre-treated with exosomes isolated from KHOS-DR/U2OS-DR cells (5 x 10^5^ in Matrigel; n = 20). Indicated mice received 1 mg/kg of Dox orally once every 3 days for 4 weeks. **(A, D)** Change in body weight of mice in the different experimental groups over 4 weeks (n=5/group). **(B, E)** The tumor growth rate in the different experimental groups (n=5/group). ****P < 0.0001, ns, not significant. **(C, F)** Kaplan-Meyer survival curve showed significantly reduced sensitivity to Dox treatment and overall survival in mice injected with KHOS/U2OS cells pre-treated with exosomes from KHOS-DR/U2OS-DR cells (n=5 per group). Of note, all mice in the vehicle group died by week 6. ****P < 0.0001. **(G, H)** KHOS/U2OS cells pre-treated with exosomes from KHOS-DR/U2OS-DR exhibited significant lung metastasis even after treatment with Dox. Shown are representative IHC staining of osteocalcin. Scale bar, 1 mm.

We next determined if the observed *in vitro* effects of exosome in inducing drug resistance will translate *in vivo*. Nude mice were injected with Matrigel (sham control) (n=10), KHOS/U2OS cells (n = 20), and KHOS/U2OS cells pre-treated with exosomes isolated from KHOS-DR/U2OS-DR cells (n = 20). Half of the mice were then administered 1 mg/kg of Dox orally once every 3 days for 4 weeks. There was no difference in body weight of mice in the different experimental groups over the duration of the experiment (**Figures 4A, D**). Tumor growth in mice injected with KHOS/U2OS cells and treated with Dox was significantly lower compared to untreated mice (**Figure 4B**). However, mice injected with KHOS/U2OS cells pre-treated with exosomes derived from KHOS-DR/U2OS-DR cells did not respond to Dox treatment, and tumor growth in these mice mimicked growth rate observed with untreated mice (**Figures 4B, E**). Significantly reduced overall survival was observed in mice injected with KHOS/U2OS cells pre-treated with exosomes from KHOS-DR/U2OS-DR cells compared to mice injected with untreated KHOS/U2OS cells (**Figures 4C, F**).

We next performed IHC analysis of osteocalcin expression in lung tissue because lung is one of the organs in which KHOS/U2OS cells metastasize in xenografts. No osteocalcin expression was detected in mice from the sham control group. Mice injected with KHOS/U2OS cells and treated with Dox showed significantly less lung metastasis compared to mice that were injected with KHOS/U2OS cells and not treated with Dox. However, mice injected with KHOS/U2OS cells pre-treated with exosomes from KHOS-DR/U2OS-DR cells showed robust lung metastasisirrespective of Dox treatment (**Figures 4G, H**). Taken together, these results showed that exosomal content from drug resistant OS cells can induce chemoresistance *in vitro* and *in vivo*.

### Exosomal lncRNA ANCR Regulates Sensitivity to Dox

**Figure 5:**
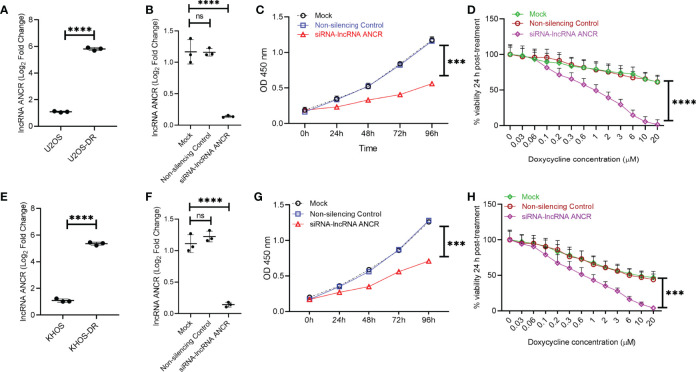
Exosomal lncRNA ANCR dictates *in vitro* sensitivity to Dox. **(A, E)** Relative expression of *DANCR* in exosomes isolated from KHOS, KHOS-DR, U2OS, and U2OS-DR cells. Data is represented as fold change relative to the respective Dox-sensitive cell type. Data is representative of three independent experiments, each measured in triplicate. Error bars, SD; ****P < 0.0001. **(B, F)** KHOS-DR/U2OS-DR cells were either mock transfected, or transfected with non-silencing control siRNA, or siRNA targeting *DANCR*. Seventy two hours post-transfection, successful knockdown of *DANCR* expression were confirmed by RT-qPCR. Data is representative of three independent experiments, each measured in triplicate. Error bars, SD; ****P < 0.0001; ns, not significant. **(C, G)** Relative growth of the KHOS-DR/U2OS-DR cells mock-transfected, or transfected with non-silencing control siRNA, or siRNA targeting *DANCR* were quantified using CCK8 assay. Knock-down of *DANCR* significantly reduced growth rate over 4 days compared to mock or non-silencing control siRNA, respectively. Data is representative of three independent experiments, each measured in triplicate. Error bars, SD; ****P < 0.0001. **(D, H)** The differently transfected KHOS-DR/U2OS-DR cells were treated with indicated concentration of Dox for 24 h and cell viability was measured. Knockdown of *DANCR* induced Dox-sensitivity in the KHOS-DR/U2OS-DR cells (IC_50_ 0.9937 ± 0.08 µM from ≥ 20 µM). Data is representative of three independent experiments, each measured in triplicate. Error bars, SD; ***P < 0.001, ****P < 0.0001.

Given that expression of the lncRNA ANCR has been shown to be critical for OS pathogenesis, we next determined the relative expression of *DANCR* (encoding lncRNA ANCR) in exosomes isolated from KHOS, KHOS-DR, U2OS, and U2OS-DR cells. Expression of *DANCR* was significantly higher in KHOS-DR and U2OS-DR cells compared to the KHOS and U2OS cells, respectively (**Figures 5A, E**). To determine if the high expression of lncRNA ANCR is important for drug resistance observed in KHOS-DR/U2OS-DR cells, we transfected these cells either with a siRNA targeting ANCR or a non-silencing control. Successful knockdown was confirmed by RT-qPCR (**Figure 5B**). Next, we determined the relative growth of the KHOS-DR/U2OS-DR cells mock-transfected, or transfected with non-silencing control siRNA, or siRNA targeting *DANCR* using the CCK8 assay. Knock-down of *DANCR* significantly reduced growth rate over 4 days compared to mock or non-silencing control siRNA, respectively (**Figures 5C, G**). We next determined if downregulation of ANCR in the KHOS-DR/U2OS-DR cells would increase their sensitivity to treatment with Dox. The differently transfected KHOS-DR/U2OS-DR cells were treated with increasing concentration of Dox for 24 h and cell viability was measured by the MTT assay. Knockdown of lncRNA ANCR significantly induced Dox-sensitivity in the KHOS-DR/U2OS-DR cells (IC_50_ 0.9937 ± 0.08 µM from ≥ 20 µM) (**Figures 5D, H**).

**Figure 6:**
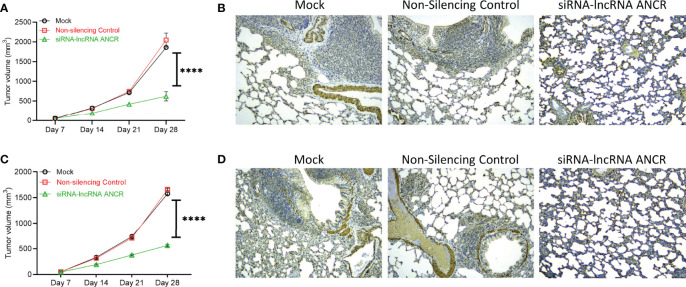
Expression of exosomal lncRNA ANCR is critical for drug resistance and OS tumor progression *in vivo*. Nude mice were injected with KHOS/U2OS cells (5 x 10^5^ in Matrigel; n = 15) pre-treated with exosomes isolated from KHOS-DR/U2OS-DR cells either mock transfected, or transfected with non-silencing control siRNA, or siRNA targeting *DANCR* and added to KHOS/U2OS cells. All mice were treated with 1 mg/kg of Dox orally once every 3 days for 4 weeks. **(A, C)** The tumor growth rate in the different experimental groups (n=5/group). ****P < 0.0001. **(B, D)** Knockdown of *DANCR* made the KHOS/U2OS cells sensitive to Dox and completely attenuated lung metastasis. Shown are representative IHC staining of osteocalcin. Scale bar, 1 mm.

We next determined if expression of exosomal lncRNA ANCR was critical for inducing drug resistance and OS tumor progression. Nude mice were injected with KHOS/U2OS cells pre-treated with exosomes isolated from KHOS-DR/U2OS-DR cells either mock transfected, or transfected with non-silencing control siRNA, or siRNA targeting *DANCR* and added to KHOS/U2OS cells. All mice were treated with 1 mg/kg of Dox orally once every 3 days for 4 weeks. Exosomes from the KHOS-DR/U2OS-DR cells in which expression of lncRNA ANCR was knocked down by ANCR siRNA failed to induce chemoresistance to Dox in mice (**Figures 6A, C**). IHC analysis of osteocalcin expression in mice injected with KHOS/U2OS cells treated with exosomes isolated from KHOS-DR/U2OS-DR cells, either mock transfected or transfected with a non-silencing control siRNA revealed robust lung metastasis (**Figures 6B, D**). However, lung metastasis was attenuated in mice injected with KHOS/U2OS cells treated with exosomes isolated from KHOS-DR/U2OS-DR cells, transfected with siRNA targeting lncRNA ANCR (**Figures 6B, D**). Cumulatively, these results indicated that the functional contribution of exosomes isolated from chemoresistant OS cells is at least in part due to the high expression of the lncRNA ANCR.

### Chemoresistance and Survival in OS Patients Is Regulated by Expression Levels of Exosomal lncRNA ANCR

**Figure 7:**
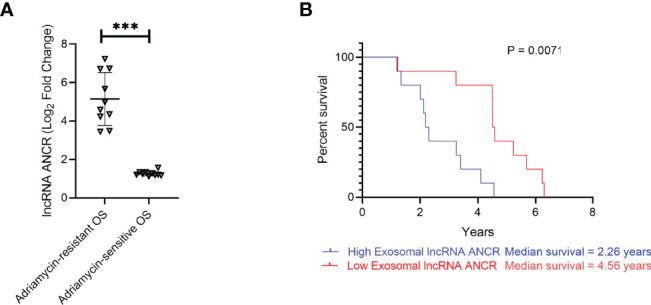
Expression of *DANCR* is correlated to resistance to Adriamycin and overall survival is patients with OS. **(A)** Expression of *DANCR* in patients with OS sensitive or resistant to Adriamycin (n=10 each) were determined using RT-qPCR. Data is representative of three technical replicates in each of the patients. Expression of lncRNA was significantly higher in OS patients resistant to Adriamycin compared to those sensitive to Adriamycin. Error bars, SD; ***P < 0.001. **(B)** Kaplan-Meyer survival curve showed significantly higher overall survival in patients with lower ANCR expression (median survival 4.56 years) compared to higher ANCR expression (median survival 2.26 years; P = 0.0071.

Expression of exosomal lncRNA ANCR was determined in serum isolated from OS patients chemosensitive or chemoresistant to Adriamycin. Expression was significantly higher in OS patients chemoresistant to Adriamycin (**Figure 7A**). Patients with lower ANCR expression and chemosensitive to Adriamycin (median survival 4.56 years) had significantly higher overall survival compared to chemoresistant OS patients with higher ANCR expression (median survival 2.26 years; P = 0.0071) (**Figure 7B**).

## Discussion

Chemotherapy regimen in OS patients comprises a combination of cisplatin, doxorubicin, high-dose methotrexate, and isofamide (39). Even though these drugs do improve disease outcome, they are not optimal due to high prevalence of chemoresistance in OS patients (40). This highlights the need to understand mechanisms driving drug resistance in OS patients.

Exosomes, membrane vesicles ranging in size from 30 – 120 nm, are secreted by multitude of human cells (41). Exosomes function as intracellular messengers effecting their regulation by their mRNA and protein content (42). It has been shown that exosomal proteins, miRNAs, circular RNAs, and lncRNAs can serve as non-invasive biomarker as well as critical regulators of different cell-cell communication and signaling pathways (43–45). Indeed, it has been shown that exosomal circular RNA can drive chemoresistance (36, 46).

The lncRNA ANCR has been shown to be important in regulation of osteoblast differentiation (47). In addition, it has been shown to be upregulated in OS patients and central to disease progression (37). The results from the current study show that exosomal expression of lncRNA ANCR is higher in chemoresistant OS patients and cells, and modulation of its expression directly impacts cell proliferation, chemosensitivity, and disease progression *in vivo*. It was thus unsurprising to find that patients with higher exosomal lncRNA ANCR expression had lower overall survival compared to patients with lower exosomal lncRNA ANCR expression.

Exosomes have been shown to be critical in tumor progression (48). Indeed, exosomes are potent drug delivery agents and can be used to transport nucleic acid and/or proteins. In fact, exosomal RNA has been shown to be a useful biomarker in OS patients based on their chemosensitivity (49). Furthermore, it has been shown that exosome-mediated delivery of P-gp and MDR-1 messenger RNA drives chemotherapy resistance in OS (50). It was shown that exosomes-derived from chemoresistant OS cells can induce Dox resistance in secondary cells (50). These results are in direct agreement to the findings from the current study.

In this study we focused entirely on plasma-derived exosomes. It will be important to determine if tumor lesion-derived exosomes from chemoresistant OS cells have similar function in driving chemoresistance and whether plasma-derived exosomes arise from tumor lesion-derived exosomes. In conclusion, our results show that lncRNA ANCR expression is upregulated in serum of chemoresistant OS patients and OS cell lines and expression is inversely correlated to overall survival in OS patients. Modulation of expression of lncRNA ANCR can be used as a therapeutic modality to induce chemosensitivity in OS cells and should be tested in additional pre-clinical models. Beyond that, the ease and non-invasive nature of isolating serum-derived exosomes should pave the way of using lncRNA ANCR as a prognostic and predictive (of chemosensitivity) biomarker in patients with osteosarcoma.

## Data Availability Statement

The original contributions presented in the study are included in the article/supplementary material. Further inquiries can be directed to the corresponding author.

## Ethics Statement

The studies involving human participants were reviewed and approved by West China Hospital. The patients/participants provided their written informed consent to participate in this study. The animal study was reviewed and approved by West China Hospital of Sichuan University.

## Author Contributions

LM designed the experiments; YW prepared the manuscript; XH, L-yT, FT, Y-yW, C-xZ, JW performed the experiments. Y-qZ and T-jG analyzed the data. XH and L-yT revised the manuscript. and all authors read the manuscript and approved the submission.

## Funding

This study was supported by the National Natural Science Foundation of China (81702664).

## Conflict of Interest

The authors declare that the research was conducted in the absence of any commercial or financial relationships that could be construed as a potential conflict of interest.

## Publisher’s Note

All claims expressed in this article are solely those of the authors and do not necessarily represent those of their affiliated organizations, or those of the publisher, the editors and the reviewers. Any product that may be evaluated in this article, or claim that may be made by its manufacturer, is not guaranteed or endorsed by the publisher.
